# Molecular differences between two Jeryl Lynn mumps virus vaccine component strains, JL5 and JL2

**DOI:** 10.1099/vir.0.013946-0

**Published:** 2009-12

**Authors:** Phil Chambers, Bert K. Rima, W. Paul Duprex

**Affiliations:** Centre for Infection and Immunity, School of Medicine, Dentistry and Biomedical Sciences, Medical Biology Centre, Queen's University Belfast, 97 Lisburn Road, Belfast BT9 7BL, Northern Ireland, UK

## Abstract

The Jeryl Lynn (JL) vaccine against mumps virus (MuV) contains two components, MuV^JL5^ and MuV^JL2^, which differ by over 400 nt. Due to the occurrence of bias in the direction of mutation, these differences and those found in nucleotide sequences of different isolates of the minor component in the vaccine (MuV^JL2^) might be due to the effect of ADAR-like deaminases on MuV grown in tissue-cultured cells. A molecular clone of MuV^JL2^ (pMuV^JL2^) and MuV^JL2^-specific helper plasmids were constructed in order to investigate molecular interactions between MuV^JL5^ and MuV^JL2^, to augment the existing molecular clone of MuV^JL5^ (pMuV^JL5^) and MuV^JL5^-specific helper plasmids. Genome and mRNA termini of MuV^JL2^ were characterized, and an unusual oligo-G insertion transcriptional editing event was detected near the F mRNA polyadenylation site of MuV^JL2^, but not of MuV^JL5^. Genes encoding glycoproteins of rMuV^JL2^ and rMuV^JL5^ have been exchanged to characterize the oligo-G insertion, which associated with the specific sequence of the F gene of MuV^JL2^ and not with any other genes or the RNA-dependent RNA polymerase of strain MuV^JL2^. The results indicate that a single G-to-A sequence change obliterates the co-transcriptional editing of the F mRNA and that this oligo-G insertion does not affect the growth of the virus.

## INTRODUCTION

The Jeryl Lynn (JL) mumps virus (MuV) vaccine contains two different component strains, MuV^JL5^ and MuV^JL2^, that differ considerably in their nucleotide sequences (Amexis *et al.*, 2002). The mechanisms that generated the two variants are unclear. MuV belongs to the genus *Rubulavirus* of the subfamily *Paramyxovirinae*. The inner core consists of the non-segmented negative-strand viral RNA (length, 15 384 nt) associated with the nucleocapsid (N) protein, lesser amounts of the polymerase associated phospho- (P) protein and small amounts of the large (L) RNA-dependent RNA polymerase (RdRp) protein. In the virion, the ribonucleoprotein (RNP) is surrounded by a host-derived membrane, which is spanned by two glycoproteins: the haemagglutinin–neuraminidase (HN) and fusion (F) proteins. On the inner face of the membrane is a membrane or matrix (M) protein that interacts with the cytoplasmic tails of the HN and F proteins and with the RNP. The virus also expresses three non-structural proteins. Two are derived from the second transcription unit, which encodes the non-structural V protein directly and, after co-transcriptional editing of the transcripts, gives rise to two other proteins, P and W. The W protein is a second non-structural protein consisting of a truncated version of V, which substitutes a short peptide of unknown function, encoded by the third potential reading frame, for the cysteine-rich tail that is characteristic of the V proteins of all members of the *Paramyxovirinae*. The third non-structural protein is a small hydrophobic (SH) protein that is derived from a transcription unit between those encoding the F and HN proteins (Elliott *et al.*, 1989; Elango *et al.*, 1989). Although some aspects of the replication of MuV have been studied at the molecular level, others have been inferred only by analogy to other members of the *Paramyxovirinae*. For example, the genome and 5′ transcriptional termini of MuV have been generally inferred from sequence comparisons rather than determined directly. The mRNA 3′ termini have been better characterized by sequencing cDNA clones generated by oligo-dT priming on MuV mRNA (Elango *et al.*, 1988). Furthermore, polyadenylation and polymerase slippage at the 3′ end of the genes and at the editing site have not been subjected to rigorous analysis, and direct proof of the inferred sequence determinants is lacking. The development of reverse-genetics systems (Amexis *et al.*, 2002; Lemon *et al.*, 2007) now allows many of the basic parameters of MuV replication to be studied in detail.

The MuV JL vaccine has been reported to be derived from a single clinical isolate (Afzal *et al.*, 1993), which was converted into a live-attenuated vaccine by passage of virus in non-human host cells. The complete nucleotide sequences of MuV^JL5^ and MuV^JL2^ have been reported (Clarke *et al.*, 2000; Amexis *et al.*, 2002) and show 414 nucleotide changes, resulting in 87 amino acid changes, between Muv^JL5^ and MuV^JL2^. This level of variation is at the same level as differences between genotypes of MuV. There are biological differences between MuV^JL5^ and MuV^JL2^. For example, MuV^JL2^ grows better in embryonated eggs than MuV^JL5^, but immunological differences have not been reported (Amexis *et al.*, 2002). Both MuV^JL5^ and MuV^JL2^ are non-neurovirulent in the rat neurovirulence test (Rubin *et al.*, 1999, 2000, 2003). Molecular differences other than the nucleotide sequences have not been investigated extensively.

## METHODS

### Virus strains and cell-culture procedures.

A549 and Vero cells were grown in Dulbecco's modified Eagle's medium (DMEM; Invitrogen) supplemented with 10 % (v/v) fetal calf serum (FCS; Invitrogen). MuV strains MuV^JL5^, MuV^JL2^ and MuV^KH^ were obtained from Dr D. Clarke (Wyeth-Lederle Vaccines, Pearl River, NY 10965, USA), Dr M. Afzal (National Institute for Biological Standards and Control, Potters Bar, London, UK) and Dr S. A. Rubin (Center for Biologics Evaluation and Research, Food and Drug Administration, Bethesda, MD, USA), respectively. Modified vaccinia virus Ankara (MVA-T7) was grown as described previously (Duprex *et al.*, 1999). For virus growth curves, Vero cells were grown in 75 cm^2^ bottles and infected at 50 % confluence at an m.o.i. of 0.01, and maintained in 10 ml DMEM/10 % (v/v) FCS. Virus titres were determined by TCID_50_ for both cell-associated and supernatant virus. Cells were scraped at the given time points into the growth medium and supernatant was separated by 5 min centrifugation at 3000 r.p.m. (1200 ***g***) (with pelleted cell-associated material resuspended in 10 ml fresh DMEM/10 % FCS). TCID_50_ titres were determined in triplicate by a 50 % end-point dilution assay in 96-well trays of Vero cells, as described previously (Duprex *et al.*, 1999).

### Bacterial strains and plasmids.

*Escherichia coli* strain DH5*α*IQ was used for routine transformations; highly competent *E. coli* strains XL blue and TOP10 were obtained from Stratagene and Invitrogen, respectively, and used where fewer transformants were expected. The full-length MuV clone pMuVFL encoding MuV^JL5^, helper plasmids encoding MuV^JL5^ N, P and L proteins and pMuVDICAT were obtained from Dr D. Clarke (Wyeth-Lederle Vaccines, Pearl River, NY 10965, USA). The cytomegalovirus (CMV) promoter-based expression plasmid pCG (Cathomen *et al.*, 1995) was used as the basis for construction of MuV^JL2^-specific helper plasmids pCG-N^JL2^, pCG-P^JL2^ and pCG-L^JL2^.

### Reagents.

Oligonucleotides were obtained from Qiagen. TRIzol reagent was from Invitrogen. Restriction enzymes, reverse transcriptase SuperScript III, high-fidelity *Taq* DNA polymerase, *Pfu* polymerase, Phusion DNA polymerase, Klenow fragment of DNA polymerase, exonuclease III and DNA ligase were obtained from New England Biolabs (NEB) or Invitrogen and used according to the manufacturers' instructions.

### Recombinant DNA manipulations.

PCR products corresponding to MuV genes were generated with unique terminal restriction-enzyme sites and cloned into suitable intermediate vectors to generate two vectors containing approximately half of the MuV^JL2^ genome. One half comprised the leader to the HN gene and was constructed in pUC18 by sequential ligations of genes from Muv^JL2^. The other half, comprising the L gene, was amplified in two overlapping segments and cloned sequentially into a modified form of the pBluescript-derived pMuV^JL5^ by deletion from N to HN, i.e. from an *Sph*I site near the start of the N gene to an *Nhe*I site near the end of the HN gene, thus replacing the L gene of MuV^JL5^ with that of MuV^JL2^. In the final stage, the leader to HN region of MuV^JL2^ from the pUC18-based intermediate was cloned into the pBluescript/MuV^JL2^ L gene intermediate to generate a full-length clone of strain MuV^JL2^. Details of oligonucleotides, enzymes etc. can be supplied on request.

The initial full-length clone of MuV^JL2^ was modified by rounds of *in vitro* mutagenesis of subgenomic clones, which were transferred to the full-length clone to insert further restriction sites at intergenic locations or to eliminate them elsewhere. Recombinant DNA operations were initially performed by standard RT-PCR, restrictions and ligations according to the manufacturers' protocols, but a simple form of ligation-independent cloning (Aslanidis & de Jong, 1990; Li & Evans, 1997) was used for most procedures after the generation of the first full-length clone of MuV^JL2^. In brief, 1–200 ng restricted vector and 1–200 ng desalted PCR product (generated using oligonucleotides with termini homologous with vector and a DNA polymerase that generates blunt-ended PCR products) for insert were incubated for 10 min at 37 °C in a final volume of 10 μl of NEB buffer 1 (or the buffer with the lowest ionic strength consistent with digestion of vector) with 10 units exonuclease III. Next 2 μl 1 M NaCl was added and exonuclease III was heat-inactivated at 75 °C for 15 min. The reaction mixture was cooled slowly from approximately 55 to 37 °C in about 1.5 h in an insulated beaker of water and transformed into competent *E. coli*. The final full-length clone of MuV^JL2^ was designated pMuV^JL2^ and has restriction sites with blunt ends or 5′ restriction overhangs engineered in all intergenic positions, internal to all viral genes except SH and spaced evenly throughout L, because exonuclease III is reliable for 3′–5′ digestion of such restriction sites. The clone of the HN gene of MuV^JL2^ to the pMuV^JL5^ vector involved cloning to an *Sgf*I site with a 3′ overhang on which exonuclease III is inactive. Klenow fragment of DNA polymerase, which has both 5′–3′ and 3′–5′ exonuclease activities, was used instead of exonuclease III for digestion of DNA during this procedure. *In vitro* mutagenesis was also performed by exonuclease III digestion of overlapping blunt-ended PCR termini generated with mutagenic oligonucleotides followed by annealing as above. Smaller plasmids (i.e. any gene except L in the initial small plasmid cloning vectors) could be mutated from a single PCR product, but longer DNAs (e.g. of the L gene half-genome clones) were generated as two overlapping pieces from the site of mutation to a site in the ampicillin-resistance gene of the vector.

### Determination of MuV RNA termini by rapid amplification of cDNA ends (RACE).

The 5′ termini of all MuV mRNAs and both genomic termini were determined by RACE after PCR using G-tailed cDNAs with a negative-sense gene-specific primer located close to the gene start for each mRNA (that for N generated two termini – that of the N mRNA and that of the antigenome; that for HN generated termini for both HN and SH) or a positive-sense primer located close to the end of the L gene for the 5′ terminus of the genome, and a common oligo-dC tailed primer as described previously (Barr *et al.*, 1994). The 3′ termini of F gene mRNAs were determined in similar fashion after PCR using oligo-dT-primed cDNAs with a positive-sense primer located near the end of the F gene and an oligo-dT-tailed primer. Nucleotide sequences of all RNA termini were determined directly from the PCR products.

### Rescue of recombinant viruses from cDNA clones.

Initial rescue by transfection of full-length and helper plasmids to MVA-T7-infected A549 cells, followed by overlay with Vero cells (Clarke *et al.*, 2000), was used for rescue of all recombinant viruses. When MuV^JL2^ helper plasmids were used, 2 μg pCG-N^JL2^, 1 μg pCG-P ^JL2^ and 0.1 μg pCG-L ^JL2^ substituted for the MuV^JL5^ analogues. Rescued recombinant viruses were verified by nucleotide sequence analysis of PCR-amplified viral genes using amplification without reverse transcription as a negative control.

## RESULTS

### Sequence variation between MuV^JL2^ and MuV^JL5^ is extensive

Numerous clone-to-clone variations were detected when the PCR products corresponding to MuV^JL2^ genes were sequenced and, in most instances, it was difficult to determine whether these were generated in the RT-PCR or whether they reflect genuine quasi-species variations in the virus population. The final consensus full-length sequence is available under GenBank accession no. FN431985. The numbers of differences between our consensus MuV^JL2^ sequence, the published consensus MuV^JL2^ sequence and the consensus sequence of strain MuV^JL5^ (Clarke *et al.*, 2000; Lemon *et al.*, 2007) are shown in Table 1. Three clusters of variations between our MuV^JL2^ sequences and those published were present and may be the result of hypermutation events, as they were all of the form where T (in the cDNA sequence) in one sequence was substituted by C in the other. One cluster is in the 3′ non-coding region of the L gene (nt 15254–15317) where there are six changes in 64 bases, of which five are T in the published MuV^JL2^, but are C in our sequence – MuV^JL5^ has T at all these positions. The second region is in the P gene (nt 2217–2323) where there are seven changes in 107 bases, of which six are C in the published MuV^JL2^, but are T in our sequence – MuV^JL5^ has T at these positions. The third region (nt 2459–2582) is also in the P gene (and was variable within our own series of clones) where there are three changes in 126 bases, all of which are T in the published MuV^JL2^ sequence and in one of our cDNA clones, but are C in two other cDNA clones – MuV^JL5^ has T at these positions. These three clusters contain 16 changes in 297 bases, a divergence of 5.4 %; this is very high compared with the remainder of these full-length sequences, where there are 35 changes in 14 987 nt, a divergence of 0.2 %, and thus contributes substantially to the overall genomic sum of 51 changes in 15 384 bases, a divergence of 0.3 %. A further potential biased mutation event that may have occurred during passage of MuV^JL2^ and MuV^JL5^ is located in the 3′ non-coding region of the N gene (nt 1804–1870) where there are nine changes between MuV^JL5^ and both MuV^JL2^ sequences, of which eight are T in both of the MuV^JL2^ sequences, but C in MuV^JL5^. A tenth change (nt 1820) in this region is present only in our MuV^JL2^ sequence, where T replaces C of the other two sequences.

### Transcription of the MuV^JL2^ genome

**Fig. 1. f1:**
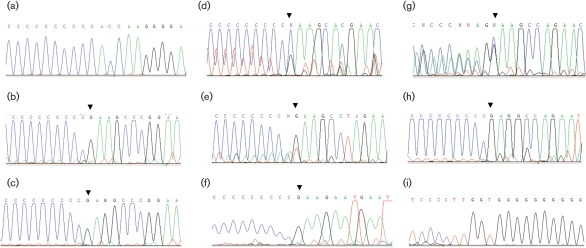
Location of MuV^JL2^ genome termini and mRNA 5′ termini. Sequence chromatograms that identify MuV^JL2^ genome termini and mRNA 5′ termini. In (a–h), the orientation of the chromatograms has been reversed so that all sequences can be read in the standard positive direction. Ten bases of tail/cap artefact and 10 bases of viral sequence are shown for each. (a, i) Genome 5′ and 3′ termini; (b–h) 5′ termini of the N, P/V, M, F, SH, HN and L mRNAs, respectively. Pairs of sequences (a) and (b) or (f) and (g) were taken from the same electrochromatograms. Arrowheads in (b–h) indicate the base inserted complementary to the mRNA methylated guanosine cap structure – this is predominantly C in the cDNA copy and thus appears as G in this orientation. The peak signals in the M mRNA start (d) were higher than optimal, generating artefact peaks under the tail. The F mRNA start (e) also contains a minor signal from the M–F gene junction. The HN mRNA start (g) contains a signal from an abundant SH–HN transcript of approximately equal intensity to the tail itself (Afzal *et al.*, 1990).

Direct determination of the MuV genome and mRNA 5′ termini by PCR and sequencing was carried out in parallel with PCR and sequencing during construction of pMuV^JL2^. This is important, as the current basis for the MuV genome annotation is founded primarily on inference. The 5′ termini of all mRNAs and the genomic termini of MuV^JL2^ were determined precisely by RACE (Fig. 1). All termini were found to be as suggested previously on the basis of sequence homology and conservation (Elango *et al.*, 1988; Okazaki *et al.*, 1992). A non-templated base [predominantly seen as G in the positive sense, as found previously (Barr *et al.*, 1994; Collins *et al.*, 1984)] was present between the mRNA 5′ termini and the cDNA tail for all mRNA starts, and corresponds to the mRNA cap structure, as no such nucleotide is present in either genome-terminal sequence.

**Fig. 2. f2:**
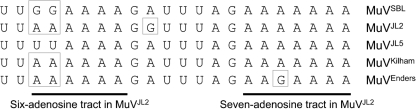
Potential polyadenylation motifs at the 3′ termini of MuV F gene transcripts. The sequences of the 3′-terminal regions (positive strand) of the F mRNAs from MuV^SBL^, MuV^JL2^, MuV^JL5^, MuV^KH^ and MuV^Enders^ are aligned, with differences from MuV^JL5^ boxed. The two polyadenosine tracts at the end of the F gene of MuV^JL2^ are indicated by bars beneath the sequences.

**Fig. 3. f3:**
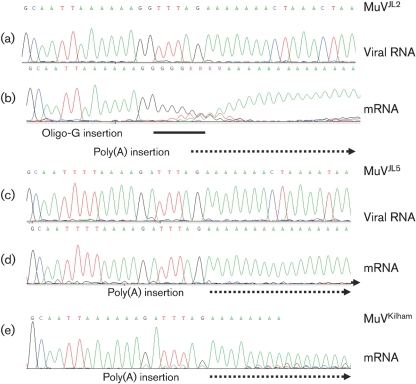
Oligo-G insertion event at the 3′ terminus of the F mRNA in MuV^JL2^. Sequence chromatograms from strains MuV^JL2^ (a, b), MuV^JL5^ (c, d) and MuV^KH^ (e) are shown. (a) and (c) show the virion RNA sequence of the F–SH intergenic region determined from PCR products generated with F- and SH-specific primers, where random primers were used to prime cDNA synthesis on virion RNA extracted from centrifuged growth medium (i.e. supernatants) from infected cells (primarily genome/antigenome RNA). (b), (d) and (e) show the 3′-terminal region of the F mRNA determined from PCR products generated with F gene- and poly(A)-specific primers, where oligo-dT was used to prime cDNA synthesis on RNA extracted from infected cells (primarily mRNA). The oligo-G insertion event in (b) is indicated by a black bar and polyadenylation in (b), (d) and (e) by dotted arrows.

In an early study (Elango *et al.*, 1988), the mRNA 3′ termini and polyadenylation signals of all genes except L were identified directly in the SBL1 strain of MuV (MuV^SBL^) and located in tracts of six and seven adenosine residues (positive-sense sequence). We directed our attention to the 3′ end of the F gene of MuV^JL2^ where there are two such tracts separated by only seven intervening bases, one of six and one of seven adenosine residues, which are both rather similar to potential polyadenylation-site motifs. There is also sequence divergence between strains MuV^JL2^ and MuV^JL5^ with three changes out of eight bases in part of the region comprising the two polyadenylation motifs (Fig. 2). In addition, the Enders strain of MuV (MuV^Enders^) expresses only F–SH read-through transcripts and has an A-to-G mutation in the region comprising the seven-adenosine tract of the other MuV strains (Takeuchi *et al.*, 1991). We mapped the 3′ end of the F mRNA by RACE to determine which polyadenylation motif (or neither, or both) might be used during mRNA synthesis for MuV^JL2^. All polyadenylation of the F gene of MuV^JL2^ occurred at the second motif (Fig. 3) in the seven-adenosine tract at the end of the F gene, which is also used for polyadenylation of the MuV^JL5^ F mRNA.

### An oligo-G insertion transcriptional editing event in the 3′ UTR of the F gene of MuV^JL2^

Although the first of the two potential polyadenylation signals did not appear to be used, it did allow slippage of the RdRp to occur as, surprisingly, a small number of additional G residues were inserted into the F mRNA in MuV^JL2^-infected cells at the GG sequence after the first motif (Fig. 3b[Fig f3]). This process resembles slippage of the viral polymerase at the P/V editing site in the P gene. The F gene of the Kilham strain of MuV (MuV^KH^) is similar to MuV^JL2^ in that it contains tracts of both six and seven adenosine residues at the end of the F gene (Tecle *et al.*, 2000; Lemon *et al.*, 2007), but is similar to MuV^JL5^ in that it contains GA instead of GG between the two tracts (Fig. 2). When the 3′-terminal region of the MuV^KH^ F mRNA was sequenced, there was no oligo-G insertion, which suggests that the presence of the second G of the GG between the two adenosine tracts is necessary for the oligo-G insertion event to occur (Fig. 3e[Fig f3]).

### Development of a reverse-genetics system for MuV^JL2^

**Fig. 4. f4:**
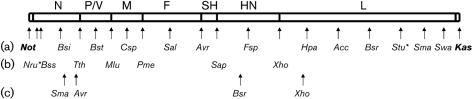
Molecular clone of MuV^JL2^, indicating gene boundaries and restriction sites in pMuV^JL2^. The bar shows the antigenome of pMuV^JL2^ and the locations of viral genes (not to scale). Arrows beneath the bar indicate the location of unique restriction sites suitable for ligation-independent cloning using exonuclease III in pMuV^JL2^. The vector sequence flanking the antigenome contains a *Not*I site upstream of a T7 RNA polymerase promoter located 5′ to the antigenome (i.e. to the left of N) and a *Kas*I site downstream of the antigenome 3′ terminus (i.e. to the right of L) which is internal to the hepatitis delta ribozyme (these restriction sites are shown in bold). (a) Restriction sites present in the consensus MuV^JL2^ sequence – these were either already unique in the consensus MuV^JL2^ sequence or made unique by mutagenesis of sites at other locations in the MuV genome or the plasmid vector. (b) Restriction sites introduced into the final clone by *in vitro* mutagenesis. Additional *Sma*I, *Avr*II, *Bsr*GI and *Xho*I restriction sites in the MuV^JL2^ sequence (c) were removed by *in vitro* mutagenesis. A *Sap*I site and two *Fsp*I sites were removed from the vector sequence by *in vitro* mutagenesis or deletion to render sites in the MuV^JL2^ sequence unique in the final clone. Restriction-enzyme names are abbreviated for clarity. Details of their position in the MuV^JL2^ sequence are available on request. The asterisks indicate that these sites are unique in the plasmid DNA which is methylated, as there are two sites at 11408–11413 and 11608–11613 that are also cleavable with *Stu*I and *Nru*I, respectively, in unmethylated plasmid DNA.

It became clear that not only was the sequence of our MuV^JL2^ component (Afzal *et al.*, 1993) (received as an early passage after isolation and plaque purification of the MuV^JL5^ and MuV^JL2^) different from the MuV^JL5^ sequence, but also that the level of variation between our MuV^JL2^ sequence and the published MuV^JL2^ sequence was significant (51 nt). In order to facilitate further molecular studies, to investigate the phenotypes of recombinants between MuV^JL2^ and MuV^JL5^, we developed a reverse-genetics system for MuV^JL2^ that complements the existing system for MuV^JL5^ (Lemon *et al.*, 2007; Clarke *et al.*, 2000). The plasmid pMuV^MPBS^ (Lemon *et al.*, 2007) derived from MuV^JL5^ is hereafter referred to as pMuV^JL5^ for clear differentiation from pMuV^JL2^. Plasmid pMuV^JL2^ was constructed by using the pBluescript plasmid, T7 promoter and hepatitis delta ribozyme from the pMuV^JL5^ system (Clarke *et al.*, 2000) and engineered to fit the consensus sequence described above except (i) where restriction sites were introduced or removed that would not alter the viral proteins, but would allow easy exchange of genes between MuV^JL5^, MuV^JL2^ and other MuV strains, and (ii) where a potential minor mutation event was detected in the P gene (three nucleotides, 2459, 2534 and 2582, are all T in the full-length plasmid, but C in most of the P gene clones), because both MuV^JL5^ and the previously published MuV^JL2^ sequence have T at these three positions. Restriction-enzyme sites at intergenic locations between genes encoding the envelope proteins of pMuV^JL5^ have been incorporated during the construction of pMuV^JL2^ (the unique restriction site between F and SH is, however, *Avr*II in pMuV^JL2^ rather than *Bmg*BI as in pMuV^JL5^, because pMuV^JL2^ has an additional *Bmg*BI site in the L gene and there are both *Sgf*I and *Sap*I unique restriction sites between SH and HN in pMuV^JL2^). Additional unique restriction-enzyme sites have been generated between all other genes and internal to all viral genes (except for that of SH) and spaced fairly evenly throughout the L gene to facilitate molecular studies (Fig. 4).

**Fig. 5. f5:**
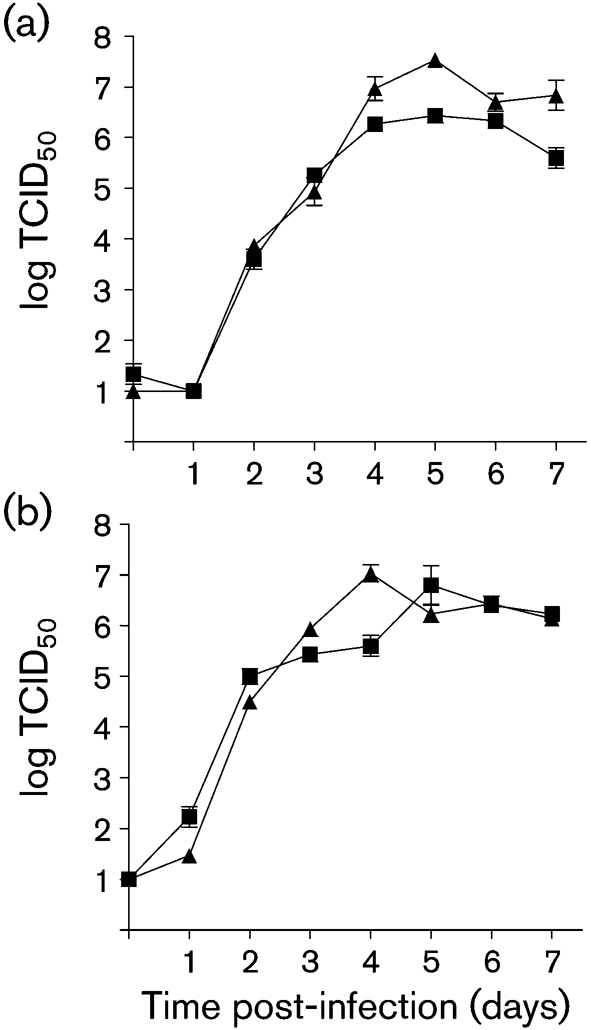
Growth curves of MuV^JL2^ and rMuV^JL2^. Supernatant (a) and cell-associated (b) titres of samples harvested from infected Vero cells at days 0–7 post-infection were determined by TCID_50_. The stock plaque-purified MuV^JL2^ supplied by Dr M. Afzal and the final recombinant virus rescued from cDNA are shown as MuV^JL2^ (▪) and rMuV^JL2^ (▴), respectively. Error bars represent sd.

Although helper plasmids appear to be interchangeable in the rescue systems for all molecular clones tested, from our direct experience, it appeared advisable to use homologous plasmids in order to avoid inter-strain recombination events driven by vaccinia virus (data not shown). Therefore, MuV^JL2^-specific helper plasmids encoding the N, P and L genes (pCG-N^JL2^, pCG-P^JL2^ and pCG-L^JL2^, respectively) were constructed in the CMV promoter-driven vector pCG and these were used successfully in the rescue of the pMuV^JL2^. A mumps minigenome construct that encodes enhanced green fluorescent protein (EGFP) (pMuV^EGFP^) has been constructed by replacing the chloramphenicol acetyltransferase open reading frame of pMuVDICAT (Clarke *et al.*, 2000) with that for EGFP. pMuV^EGFP^ has been rescued with both the MuV^JL2^ and MuV^JL5^ sets of helper plasmids (data not shown). A recombinant virus, rMuV^JL2^, was rescued successfully from plasmid pMuV^JL2^. The growth of this virus was very similar to that of the original plaque-purified virus MuV^JL2^, confirming that the synonymous genetic alterations that we have made do not affect virus growth dynamics in Vero cells (Fig. 5).

### Construction of chimaeric rMuV^JL2^ and rMuV^JL5^ viruses to characterize the oligo-G insertion at the end of the F mRNA of MuV^JL2^

**Fig. 6. f6:**
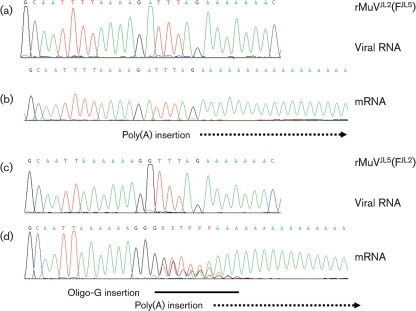
3′-Terminal sequences of F genes and transcripts of rMuV^JL2^(F^JL5^) and rMuV^JL5^(F^JL2^). (a, b) Sequence from rMuV^JL2^(F^JL5^); (c, d) sequence from rMuV^JL5^(F^JL2^). (a) and (c) show sequence electrochromatograms of the virion sequence of the F polyadenylation region cut to the first nucleotide difference between MuV^JL2^ and MuV^JL5^ in the SH gene; (b) and (d) show the 3′-terminal region of the F mRNA 2. rMuV^JL2^(F^JL5^) has the F sequence characteristic of rMuV^JL5^ and the SH sequence characteristic of rMuV^JL2^, and rMuV^JL5^(F^JL2^) has the F sequence characteristic of rMuV^JL2^ and the SH sequence characteristic of rMuV^JL5^. The oligo-G insertion event in (d) is indicated by a black bar and polyadenylation in (b) and (d) by a dotted arrow.

In order to determine whether the oligo-G insertion at the end of the F mRNA of MuV^JL2^ was determined by the nucleotide sequence at the end of the MuV^JL2^ F gene, by characteristics of the viral polymerase of strain MuV^JL2^ or by other determinants in the genome of this MuV strain, the F gene of MuV^JL2^ was transferred into MuV^JL5^ and the F gene of MuV^JL5^ into MuV^JL2^ by using the unique between-gene restriction sites that are present in the plasmids. The corresponding viruses were rescued and the 3′ termini of the F mRNAs were characterized. The recombinant rMuV^JL5^ (F^JL2^) displayed the same oligo-G insertion as the MuV^JL2^ F gene, and the rMuV^JL2^ (F^JL5^) displayed no oligo-G insertion (Fig. 6). This indicates that the oligo-G insertion was associated with the F gene sequence of MuV^JL2^ rather than being a characteristic of the viral polymerase of strain MuV^JL2^.

## DISCUSSION

The origin of the sequence variation between the MuV^JL5^ and MuV^JL2^ components of the live-attenuated JL vaccine is unclear. The JL vaccine was derived from a single clinical isolate. These, in general, display a consensus sequence with little detectable variation and, hence, it is likely that some part of the variation between the two vaccine components has been generated after the original isolation and during the attenuation process. Selection of a more neurovirulent variant of MuV^JL5^ has been shown to be associated with only three amino acid changes and relative sequence stability of this virus (Rubin *et al.*, 2003). The substantial sequence differences between MuV^JL2^ determined in different laboratories suggest that cytosine or adenine deamination events may play a significant role in the generation of diversity between the MuV^JL^ strains. It is to be noted that Amexis *et al.* (2002) did not succeed in propagating a MuV^JL2^ virus, but that the sequence was derived from sequence variations found in the vaccine. Though it was inferred to be preferably propagated in chicken embryo cells, their MuV^JL2^ could not be propagated on Vero cells and, hence, they suggested that MuV^JL2^ was a not completely defective satellite virus. Our sequence is derived from a plaque-purified Vero cell-propagated MuV^JL2^ isolate. Amexis *et al.* (2002) posed the question of whether the MuV^JL2^ sequence is a passenger virus of MuV^JL5^. The fact that we were able to set up a rescue system based on the MuV^JL2^ consensus sequence with its own helper plasmids indicates that the vaccine is a mixture of two independently replicating viruses. In fact, titres of MuV^JL2^ are routinely at least 1 log_10_ higher than those of MuV^JL5^ in Vero cells in our hands. This, and the number and location of unique restriction-enzyme sites that did not affect the viral protein sequences and the even spacing of unique restriction sites in pMuV^JL2^, should be of benefit in future molecular studies of MuV and may prove to be advantageous in the use of the MuV^JL2^ rescue system.

The prevalence of C→U and U→C mutations and the identification of the localized nature of the biased hypermutation indicate that deamination reactions by ADAR- or APOBEC-like enzymes may play a role in the generation of the sequence variation between MuV^JL5^ and MuV^JL2^, although this remains to be formally proven. In most cases, biased hypermutation events lead to functional impairment of the affected region of the viral genome, for example in measles virus sequences obtained from cases of subacute sclerosing panencephalitis (Cattaneo *et al.*, 1988). Interestingly, loss of function does not seem to be the case here. The potential events in L and N genes are located in the 3′ non-coding regions. One potential event in the P gene affects an area with low sequence similarity at the N-terminal part of the V/P protein. The other event present in most but not all of our MuV^JL2^ P clones affects the V but not the P protein and this may alter the interferon sensitivity of the MuV^JL2^ virus. Biased hypermutation has been described for several other viruses (Bass, 2002) and may reflect the deaminating activity of the ADAR or APOBEC enzymes themselves (Bass, 2002; Bishop *et al.*, 2004) or their ability to bind to RNA (Nie *et al.*, 2007).

This study shows for the first time, to our knowledge, the MuV genome and 5′ termini of the mRNAs and that these had been predicted correctly from the conservation of motifs, and demonstrates the capped nature of these mRNAs in contrast to the uncapped terminal sequences of the genome. Earlier studies were not able to determine the precise mRNA start sites, although polyadenylation signals had been identified correctly (Elango *et al.*, 1988). The polyadenylation signal at the end of the F gene of MuV^JL2^ required clarification because of the sequence variation amongst strains of MuV at this point. Read-through from the F to SH transcription units is found in MuV^Enders^ (Afzal *et al.*, 1990; Takeuchi *et al.*, 1991), where there is a failure to terminate and polyadenylate. In addition, there was sequence variation amongst our own six PCR-derived clones from virion RNA of strain MuV^JL2^ at this point – one had a single addition to the six-adenosine tract, another a single deletion from the seven-adenosine tract. It was not clear, therefore, which motif would be used as the polyadenylation signal during F gene mRNA synthesis for strain MuV^JL2^. The data showed clearly that termination and polyadenylation of the F mRNA of MuV^JL2^ occurs only at the seven-adenosine tract, as in MuV^SBL^, MuV^JL5^ and MuV^KH^. During the study of the polyadenylation signals of the F gene, an oligo-G insertion event was identified in the 3′ non-coding region of the F gene of MuV^JL2^ (and also in rMuV^JL2^). This leads to variable levels of insertion of a number of additional G residues (one to seven, with a mean of approximately four residues) at a site that directly follows the six-adenosine tract and is similar in sequence to a polyadenylation motif. The insertion of variable numbers of G residues does not appear to affect the ability of the virus to grow, and the motif appears only to be recognized during transcription and not replication of the viral genome. Exchanges of the F genes of rMuV^JL5^ and rMuV^JL2^ showed clearly that the insertion of G residues is a property of the sequence motif in the F gene of MuV^JL2^ and not of the RdRp or the other genes of MuV^JL2^, as it occurred in the F gene of MuV^JL2^ placed in either the rMuV^JL2^ or the rMuV^JL5^ background. However, the oligo-G insertion into the F mRNA prior to polyadenylation was not observed in MuV^KH^, which also contains the same six- and seven-adenosine tracts as MuV^JL2^. MuV^JL2^ has the sequence GG at the oligo-G insertion site, whereas MuV^KH^ has the sequence GA. This suggests that presence of a pair of G residues between the two polyadenosine tracts is necessary for the oligo-G insertion event to occur in MuV^JL2^. The oligo-G insertion at the 3′ end of the MuV^JL2^ F mRNA clearly resembles the viral RdRp slippage motif at the P/V editing site (Elliott *et al.*, 1990; Paterson & Lamb, 1990). At present, we do not know whether this oligo-G insertion is a chance event that reflects the conjunction of the pair of G residues between the six- and seven-adenosine tracts so far restricted to MuV^JL2^, or whether it has a function. It may, for example, regulate transcription of the SH gene by affecting transcription attenuation or the formation of read-through products, which is a frequent occurrence at this site (Afzal *et al.* 1990).

In conclusion, we have demonstrated significant sequence variation and instability in the MuV^JL2^ component of the JL vaccine. In part, these sequence changes may be generated by activity of cytosine and/or adenine deaminases in the cell. However, the inter-relationship of MuV^JL2^ and MuV^JL5^ is not clear and the sequence diversity even between isolates of MuV^JL2^ raises further questions about the relationships between the two vaccine components. We have developed a versatile rescue system for MuV based on MuV^JL2^ and used this and the MuV^JL5^ rescue system to prove that an oligo-G insertion event at the end of the F gene of MuV^JL2^ is determined by the sequence at the slippage site.

## Figures and Tables

**Table 1. t1:** Nucleotide and amino acid differences between MuV^JL5^ and MuV^JL2^ Values are shown as number of changes (percentage difference). MuV^JL2^ db is the sequence from Amexis *et al.* (2002); MuV^JL2^ cons is our consensus sequence.

**Sequence**	**Length (aa)**	**Comparison**		
		**MuV^JL5^/MuV^JL2^ db**	**MuV^JL5^/MuV^JL2^ cons**	**MuV^JL2^ db/MuV^JL2^ cons**
Nucleotide	–	414 (2.7)	421 (2.7)	51 (0.3)
Protein				
N	550	7 (1.3)	6 (1.1)	1 (0.2)
P	392	12 (3.0)	10 (2.6)	6 (1.5)
V	225	7 (3.1)	4 (1.8)	3 (1.3)
M	376	5 (1.3)	7 (1.9)	4 (1.1)
F	539	13 (2.4)	13 (2.4)	2 (0.3)
SH	58	6 (10.3)	5 (8.6)	1 (1.7)
HN	583	14 (2.4)	16 (2.7)	2 (0.3)
L	2262	13 (0.6)	10 (0.4)	3 (0.1)
